# Ship Trajectory Generator under the Interference of Wind, Current and Waves

**DOI:** 10.3390/s22239395

**Published:** 2022-12-01

**Authors:** Xian Ding, Hongwei Bian, Heng Ma, Rongying Wang

**Affiliations:** The Department of Navigation Engineering, Naval University of Engineering, Wuhan 430033, China

**Keywords:** trajectory generator, ship motion, wind, current and wave, ANFIS, inertial navigation system

## Abstract

In view of the low accuracy of the motion parameters generated by the typical ship trajectory generator, and the fact that the problem of wind, current and wave interference is not considered, this paper establishes a new ship trajectory generator by analyzing the changes in the ship’s attitude and speed under different motion states. Through simulation, the accuracy of the main motion parameters is significantly improved compared with the typical trajectory generator; the time-varying non-uniform wind, current and wave fields are constructed, and the interference effect of wind, current and waves on ship motion is analyzed by combining the empirical formulas of force and moment; an adaptive neuro fuzzy inference system (ANFIS) based on wind, current and wave interference is designed, and the fuzzy rules of the fuzzy system are determined by training and testing the measured data; the motion parameters of superimposed wind, current and wave interference are compared with the measured data, and the accuracy is further improved after superimposing wind, current and wave interference.

## 1. Introduction

A trajectory generator is a tool used to generate sensor group simulation data and corresponding navigation parameters required for the simulation of an inertial navigation system (INS) and its integrated navigation system. In the algorithm research and simulation verification of INS and its integrated navigation, the research and application of trajectory generators is indispensable. The principle is to generate the motion parameters required for the simulation test of the inertial measurement unit (IMU) by simulating the maneuvering state of the carrier [1,2,3,4]. The motion of the ship is not only affected by the maneuvering, but it also subject to interference by the sea conditions, such as wind, current and waves. In order to better study the algorithm of the ship strapdown inertial navigation system (SINS) and verify the accuracy of the algorithm by simulation, the ship trajectory generator needs to be able to generate motion parameters that conform to the ship’s motion characteristics and consider the interference of wind, current and wave.

**Figure 1:**
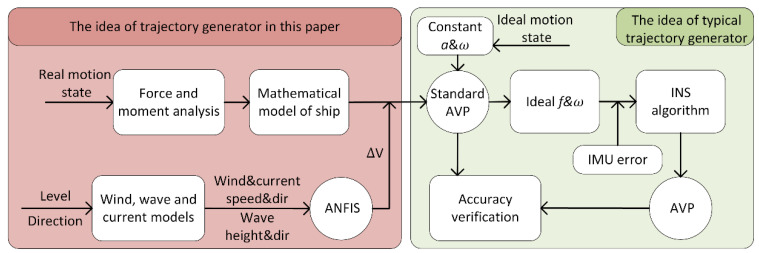
Flow chart of design and simulation of ship trajectory generator.

At present, the research objects of the trajectory generator are mainly aircraft [5,6]. The main idea of a typical trajectory generator is to calculate the acceleration and angular velocity of the carrier by presetting ideal motion states, such as uniform linear motion, uniform rotation motion, uniform acceleration motion, etc., and then calculate the attitude, velocity and position of the carrier. The main idea is shown in the green part of Figure 1. The classical example is PROFGEN, designed by the American Air Force Avionics Laboratory [7,8]. This trajectory generator can generate attitude, velocity and position information by inputting the initial state, acceleration, angular velocity and other information. Due to the preset acceleration and angular velocity information, the generated trajectory is too ideal and does not conform to the motion characteristics of the ship, so it is difficult to meet the requirements of high-precision ship SINS simulation tests. The ship trajectory generator designed in reference [9,10] takes into account the characteristics of the ship, but it is still based on the ideal motion states, such as uniform circumference and uniform acceleration, and cannot generate complex ship trajectories. In reference [11,12], the ship motion parameter generator designed on the basis of ship measured data can generate a relatively complex ship motion trajectory, but it does not consider the interference of wind, current and waves. In the study of wind, current and waves, a variety of calculation formulas for wind, current and wave interference forces and moments have been proposed [13,14]. For example, Wu et al. [15], Zhao Qiaosheng [16] and Min Guk Seo [17] have, respectively, studied the empirical formulas for wind, current and wave forces and moments. However, the described formulas involve complicated hydrodynamic parameters and detailed ship type data, which are usually difficult to obtain and cannot be directly applied to the ship trajectory generator.

Aiming at the above problems, this paper designs a new ship trajectory generator under wind, current and wave interference. Compared with the typical trajectory generator, the main changes are as shown in the red area in Figure 1. The main ideas are as follows: (1) based on the motion state, the ship motion characteristics are analyzed, and the ship trajectory generator without wind, current and wave interference is established; (2) the time-varying wind, current and wave field is established to analyze the interference of wind, current and waves in the ship motion parameters; (3) an adaptive neuro fuzzy inference system (ANFIS) is designed; with the wind and wave information of the measured data as the input and the speed change as the output, the ANFIS rules are determined through neural network training; (4) the ship trajectory generator after superposing wind, current and wave interference is compared with the measured data to verify the rationality and superiority of ANFIS.

## 2. Mathematical Model of Ship Motion

**Figure 2:**
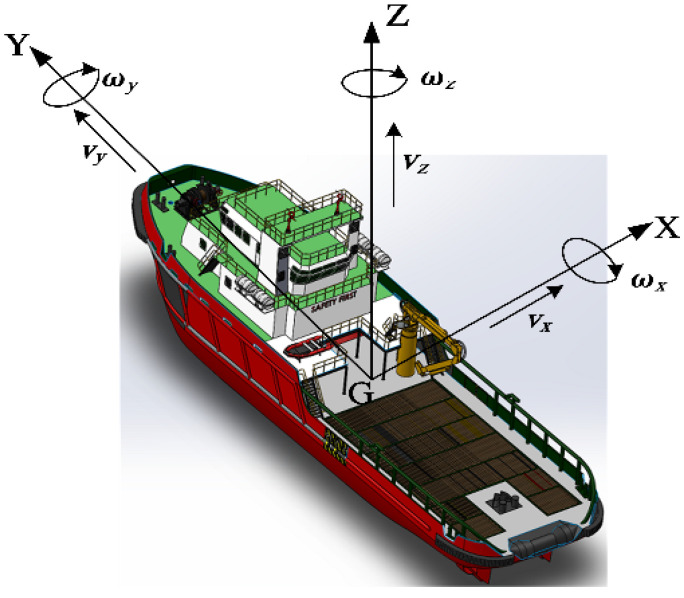
Schematic diagram of ship motion variables.

The ship motion states can be divided into uniform linear motion, variable speed motion and steering motion. In order to simulate the ship characteristics, it is necessary to focus on the acceleration and angular velocity change analysis of variable speed motion and steering motion. The description of the ship motion variables in the carrier coordinate system is shown in Figure 2.

(1) Uniform linear motion

Without wind, current and wave interference, the acceleration and angular velocity of the ship are approximately zero when the ship sails at a constant speed.

(2) Variable speed linear motion

When there is no interference of wind, current and waves, the attitude of the straight sailing ship with variable speed will not alter, so the angular velocity can be regarded as zero. It can be expressed as ${\left(\omega_{x},\omega_{y},\omega_{z}\right)}^{T}=0$. The variable speed motion of the ship is mainly achieved by the maneuvering propeller, and the differential equation of its motion can be expressed as [18]

(1) $$m{\dot{v}}_{y}=F_{P}-R_{u}v_{y}+mv_{x}\omega_{z}$$

where *F_P_* is the propulsion force and *R_u_* is the drag coefficient along the *Y*-axis. *F_P_* can be expressed as [19]

(2) $$\left\{ \begin{array}{l} F_{P}=(1-t_{P})\rho n^{2}D_{P}{}^{4}K_{T}(J_{P})\\ J_{P}=(1-v_{z}\omega_{y})\cdot v_{y}/(n\cdot D_{P})\\ K_{T}(J_{P})=a_{0}+a_{1}J_{P} \end{array}\right.$$

Inserting *F_P_* from Equation (2) into Equation (1), Equation (1) can be expressed as

(3) $$m{\dot{v}}_{y}+\left[R_{u}-a_{1}(1-v_{z}\omega_{y})(1-t_{P})\rho n^{2}D_{P}{}^{4}/(nD_{P})\right]v_{y}=(1-t_{P})\rho n^{2}D_{P}{}^{4}a_{0}$$

where *t_p_* is the thrust reduction coefficient, *n* is the rotational speed of the propeller and *D_p_* is the propeller diameter. When the ship data are fixed, Equation (3) can be simplified as follows:

(4) $$m{\dot{v}}_{y}+Q\left(n\right)v_{y}=G(n)$$

According to the actual situation, when the ship is in a stable state at different gears, the rotational speed of the propeller is approximately a constant value. When *n* is a constant value, the speed variations of the ship reflect a first-order system. Considering the change in the rotational speed of the propeller when the ship is accelerating, combined with the measured data, this paper adopts the second-order overdamping function as the longitudinal speed model of the ship when the ship is accelerating. This is because the first-order system can be regarded as a special case of the second-order system, and the second-order system can also reflect the characteristics of the higher-order system.

Assume that the speed of the ship at the initial moment is *v*_0_, and the speed increases by Δ*v* after the propeller gear is switched; thus, the speed change can be expressed as

(5) $$v_{y}(t)=v_{0}+\Delta V(1+\frac{e^{-t/T_{1}}}{T_{2}/T_{1}-1}+\frac{e^{-t/T_{2}}}{T_{1}/T_{2}-1})$$

In (5), $T_{1}=\frac{1}{\omega_{1}(\xi_{1}-\sqrt{\xi_{1}{}^{2}-1})}$, $T_{2}=\frac{1}{\omega_{1}(\xi_{1}+\sqrt{\xi_{1}^{2}-1})}$; $\xi_{1}$ and $\omega_{1}$ are the damping ratio and oscillating frequency, respectively. By identifying the measured data, different $\xi_{1}$ and $\omega_{1}$ can be obtained, which can reflect the acceleration performance of ships in different gears [20]. Deceleration is regarded as the reverse process of acceleration and will not be analyzed in detail.

(3) Steering motion

The amplitude of pitch and heave caused by the steering motion of a ship in still water is very small, and the pitch angular velocity *ω_x_* and heave acceleration *a*_z_ are approximately zero.

According to the transfer function of the rudder angle *δ* and heading angular velocity *ω_z_* in the Nomoto response model [21],

(6) $$\frac{\omega_{z}(s)}{\delta (s)}=\frac{K(1+T_{3}s)}{\left(1+T_{1}s)(1+T_{2}s\right)}$$

Since *T*_3_ is very small and the zero point $s_{3}=-\frac{1}{T_{3}}$ is far away from the negative half axis, the removal of this point has little impact, so the heading angular velocity can be expressed as

(7) $$\omega_{z}(t)=K\delta (t)(1+\frac{e^{-t/T_{3}}}{T_{4}/T_{3}-1}+\frac{e^{-t/T_{4}}}{T_{3}/T_{4}-1})$$

In (7), the definition of *T*_3_ and *T*_4_ refers to Equation (5).

The steering movement can be divided into three stages: initial steering, constant steering and steering recovery. Assuming that the roll angle in the first stage will increase to the angle *γ*, the change in roll angle can be expressed as

(8) $$\theta (t)=\gamma (1-\frac{1}{\sqrt{1-\xi_{2}{}^{2}}}e^{-\xi_{2}\omega_{2}t}\sin(\omega_{2}\sqrt{1-\xi_{2}{}^{2}}t+\beta_{1}))$$

In (8), $\xi_{2}$ and $\omega_{2}$ are the damping ratio and oscillating frequency, respectively; *β*_1_ is the initial phase. In the phase of steady steering, the roll angle remains unchanged, so the angular velocity *ω_y_* is zero. In the phase of steering recovery, the change in roll angle can be regarded as the reverse process of the initial phase.

The displacement and attitude angle are denoted as $\mu ={\left(x,y,z,\varphi ,\theta ,\psi \right)}^{T}$, and the velocity and angular velocity are denoted as $\tau ={\left(v_{x},v_{y},v_{z},\omega_{x},\omega_{y},\omega_{z}\right)}^{T}$. The trajectory parameters of the ship can be obtained from the following formula:

(9) $$\dot{\mu }=\left[\begin{array}{cc} c_{b}^{n} & 0_{3*3}\\ 0_{3*3} & J \end{array}\right]\tau$$

In (9), $c_{b}^{n}=\left[\begin{array}{ccc} \cos\psi \cos\theta & -\sin\psi \cos\varphi +\cos\psi \sin\theta \sin\varphi & \sin\psi \sin\varphi +\cos\psi \cos\varphi \sin\theta \\ \sin\psi \cos\theta & \sin\psi \cos\varphi +\sin\psi \sin\theta \sin\varphi & -\cos\psi \sin\varphi +\sin\psi \cos\varphi \sin\theta \\ -\sin\theta & \cos\theta \sin\varphi & \cos\theta \cos\varphi \end{array}\right]$, $J=\left[\begin{array}{ccc} 1 & \sin\varphi \tan\theta & \cos\varphi \tan\theta \\ 0 & \cos\varphi & -\sin\varphi \\ 0 & \sin\varphi \sec\theta & \cos\varphi \sec\theta \end{array}\right]$.

In order to verify the feasibility and superiority of the above model, the simulation verification is carried out based on the ship’s measured data in a period of relatively calm sea conditions. The ship’s initial information and motion state are shown in Table 1 and Table 2.

**Figure 3:**
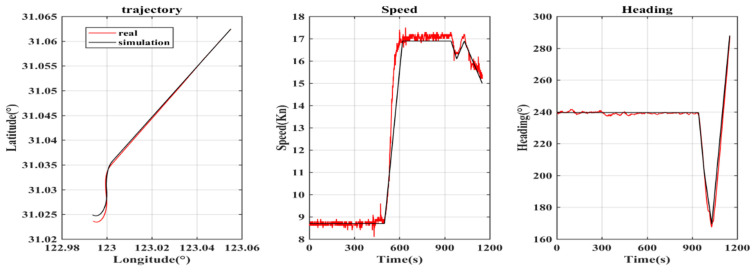
Comparison between measured data and typical trajectory generator.

**Figure 4:**
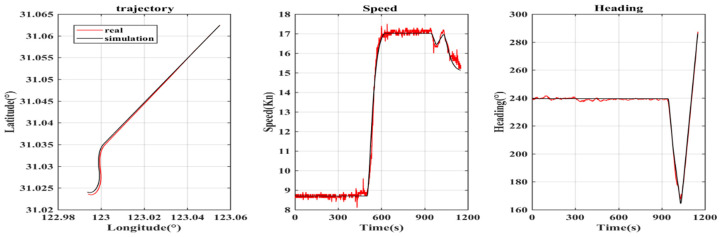
Comparison between measured data and the new trajectory generator.

Based on the position, speed and heading of the measured data, the comparison results of typical trajectory generators and the trajectory generator described in this paper are shown in Figure 3 and Figure 4. The black line in the figure denotes the data generated by the trajectory generator, and the red line denotes the measured data.

In order to judge the accuracy of the simulation results more intuitively and verify the superiority of this model, the accuracy of position, speed and heading and root-mean-square error (RMSE) are calculated. The results are shown in Table 3.

Accuracy refers to the accuracy within the acceptable error range (threshold), and RMSE reflects the statistical rule of error. It can be seen from Table 3 that the accuracy of the position, speed and heading generated by the trajectory generator described in this paper within the threshold is higher than that of the typical trajectory generator, and the RMSE value is also smaller. This is because the preset ideal track of the typical trajectory generator does not conform to the motion characteristics of the ship, which also shows that the ship trajectory generator described in this paper is superior to the typical trajectory generator.

Although the simulation effect of the model proposed in this paper is better than that of the typical trajectory generator, because the model is not disturbed by wind, current and waves, it is still quite different from real ship motion. In order to obtain more realistic ship motion parameters, it is necessary to analyze the wind, current and wave interference.

## 3. Analysis of Wind, Current and Wave Interference

In the actual navigation of a ship, the level and direction of the wind, waves and currents are constantly changing at random. It is difficult to directly address the variation in the motion parameters due to the combined interference of wind, current and waves at all times. In order to simulate the actual environment of ship navigation, it is necessary to develop time-varying inhomogeneous wind, current and wave field models that conform to the characteristics of the actual sea conditions.

### 3.1. Wind Field Model

Taking the combined wind speed model as the research object, the actual wind speed at sea is divided into three components: average wind, gradual wind and random wind [22].

#### 3.1.1. Average Wind

This component reflects the average wind speed of the wind field, which generally does not change with time and can be expressed as

(10) $$V_{m}=\frac{1}{2}(V_{i\max}+V_{i\min})$$

where *i* represents the wind level.

#### 3.1.2. Gradual Wind

Gradual wind is generally simulated by the frequency density spectrum. In this paper, the frequency spectrum of the NORSOK wind model is used, and its expression is as follows [23]:

(11) $$\left\{ \begin{array}{l} S_{w}(\omega )=320\cdot {\left(V_{m}/10\right)}^{2}\cdot {\left(z_{cw}/10\right)}^{0.45}/{\left(1+x^{0.468}\right)}^{3.561}\\ x=172\cdot \omega {\left(z_{cw}/10\right)}^{2/3}{\left(V_{m}/10\right)}^{-0.75} \end{array}\right.$$

where *V_m_* is the wind speed; *Z_cw_* is the vertical height from the wind action point to the sea level; *ω* is the frequency. The gradual wind is regarded as the result of the superposition of multiple simple harmonic waves, so the gradual wind can be expressed as

(12) $$V_{di}(t)=\sqrt{2S_{w}(\omega_{i})△\omega_{i}}\cos(2\pi \omega_{i}t+\varphi_{i})$$

where *ω_i_* is the frequency of the simple harmonic component; $△\omega_{i}$ is the frequency width of the simple harmonic component; *φ_i_* is the random phase.

#### 3.1.3. Random Wind

Random wind mainly reflects the randomness of wind in the wind field, which can be expressed by the superposition of the average wind with the first-order Markov process.

(13) $$\left\{ \begin{array}{l} {\dot{V}}_{m}+a_{1}V_{m}=w_{1}\\ {\dot{\psi }}_{w}+a_{2}\psi_{w}=w_{2} \end{array}\right.$$

where *a*_1_ and *a*_2_ are constants greater than zero; *w*_1_ and *w*_2_ are white noise.

To sum up, the wind model can be expressed as

(14) $$V_{w}=V_{m}+\sum_{i=1}^{N}V_{di}$$

**Figure 5:**
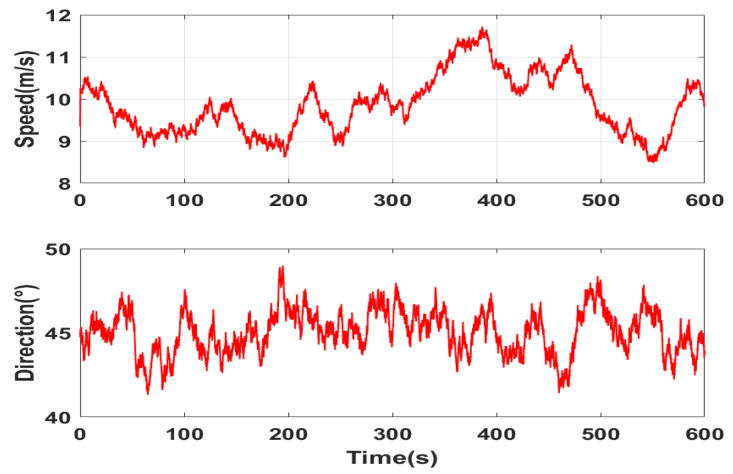
Simulation diagram of wind speed and direction.

In 10 min, the simulation of the wind field is as shown in Figure 5 when the wind speed is level 5 (8–10.7 m/s) and the average wind direction is 45°.

### 3.2. Current Field Model

Similar to the wind field, the current speed and direction also change with time and space. In order to reflect this change, the first-order Markov process can be used to express the change in current. Its expression is similar to that in Equation (11) and will not be detailed here.

**Figure 6:**
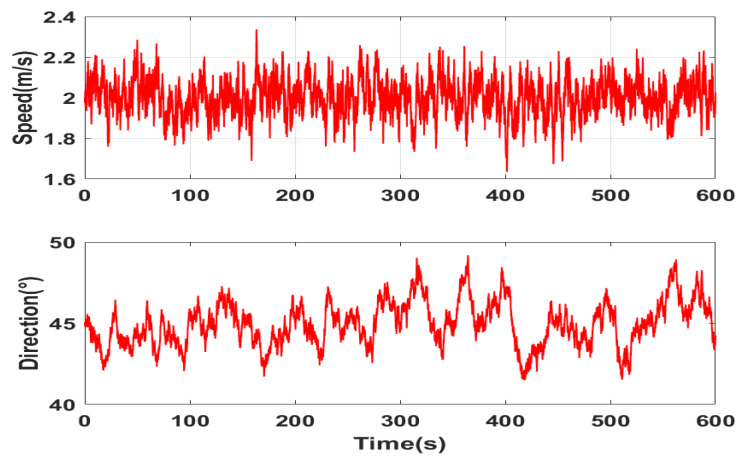
Simulation diagram of current speed and direction.

In 10 min, the simulation of the current velocity is 2 m/s and the current direction is 45°, as shown in Figure 6.

### 3.3. Wave Field Model

Generally, in practical applications, the wave energy spectrum is used to describe irregular waves, such as the PM spectrum, ITTC/ISSC spectrum, JONSWAP spectrum, etc. In order to reduce the unknown parameters, the ITTC single-parameter wave energy spectrum is adopted in this paper, which can be expressed as [24]

(15) $$S(\omega )=\frac{A}{\omega^{5}}\exp(-B/\omega^{4})$$

where *A* = 8.1 ∗ 10^−3^ g^2^; *B* = 3.11/*h*^2^; *ω* is the wave frequency; *h* is the significant wave height, which can be obtained from the wave level. The wave can be expressed as

(16) $$\xi_{wa}=\sqrt{2S_{wa}(\omega_{p})△\omega_{p}}\cos(\omega_{p}t+\varphi_{p})$$

**Figure 7:**
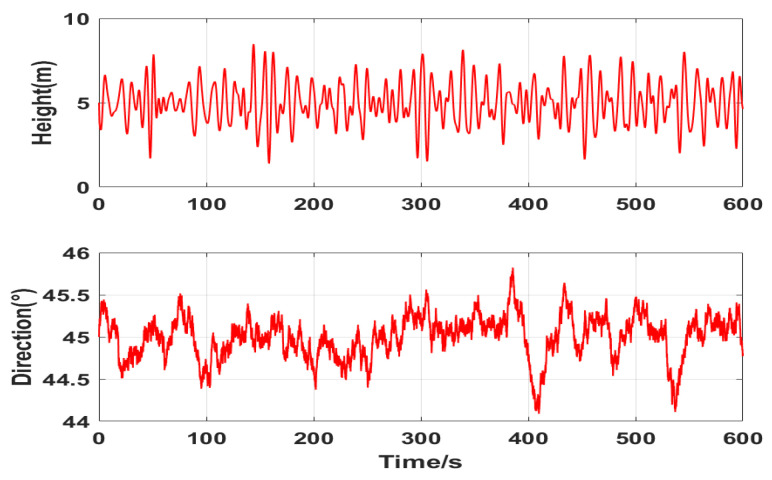
Simulation diagram of wave speed and direction.

Waves are generally caused by wind, and the average wave direction is consistent with the average wind direction. In 10 min, the wave height and wave direction simulation is as shown in Figure 7 when the wave is level 6 and the average wave direction is 45°.

### 3.4. Wind, Current and Wave Interference Analysis

Generally, compared with the waves, the wind has less influence on the vertical linear acceleration and pitch angular velocity of the ship, so the vertical velocity *a_z_* and pitch angle velocity *ω_x_* caused by it can be regarded as zero. Combined with the empirical formula of the wind force and moment, the change in the ship’s linear acceleration and angular velocity caused by wind can be simplified as [25]

(17) $$\left\{ \begin{array}{l} a_{y}=K_{1}v_{rw}{}^{2}\cos\chi_{rw}\\ a_{x}=K_{2}v_{rw}{}^{2}\sin\chi_{rw}\\ \omega_{z}=K_{3}v_{rw}{}^{2}\sin\chi_{rw} \end{array}\right.$$

where *K*_1_, *K*_2_ and *K*_3_ are wind-related proportional coefficients; *v*_rw_ is the relative wind speed; *χ_rw_* is the relative wind direction angle.

It can be seen from the characteristics of the current that it has little influence on the ship’s angular velocity. The interference of the current mainly exerts an influence on the longitudinal and transverse linear velocity of the ship. Assuming that the velocity of the current is *V*_c_, the direction is *γ*_c_, the ship’s heading is *ψ* and the ship’s longitudinal and transverse speeds are *V*_y_ and *V*_x_, respectively, and the method of speed vector synthesis is adopted, the ship’s speed after the superimposed current interference is

(18) $$\left\{ \begin{array}{l} V_{yc}=V_{y}+V_{c}\cos(\pi -\gamma_{c}-\psi )\\ V_{xc}=V_{x}+V_{c}\sin(\pi -\gamma_{c}-\psi ) \end{array}\right.$$

When the length of the ship is far less than the wave length, combined with the wave force and moment formula and the wave surface attitude, the wave interference can be simplified as follows [26]:

(19) $$\left\{ \begin{array}{l} a_{y}=K_{4}h^{2}\cos\chi_{wa}\\ a_{x}=K_{5}h^{2}\sin\chi_{wa}\\ a_{z}=\dot{h}(t)\\ \omega_{y}=0\\ \omega_{x}=\frac{\dot{h}(t)}{\sqrt{L^{2}-{\left(h(t)\right)}^{2}}}\\ \omega_{z}=K_{6}h^{2}\sin\chi_{wa} \end{array}\right.$$

where *K*_4_, *K*_5_ and *K*_6_ are the wave-related proportional coefficients; *L* is the length of the ship; h is the wave amplitude of the wave, and its value is obtained from the wave field; *χ*_wa_ is the relative wave direction angle.

### 3.5. Design of ANFIS

**Figure 8:**
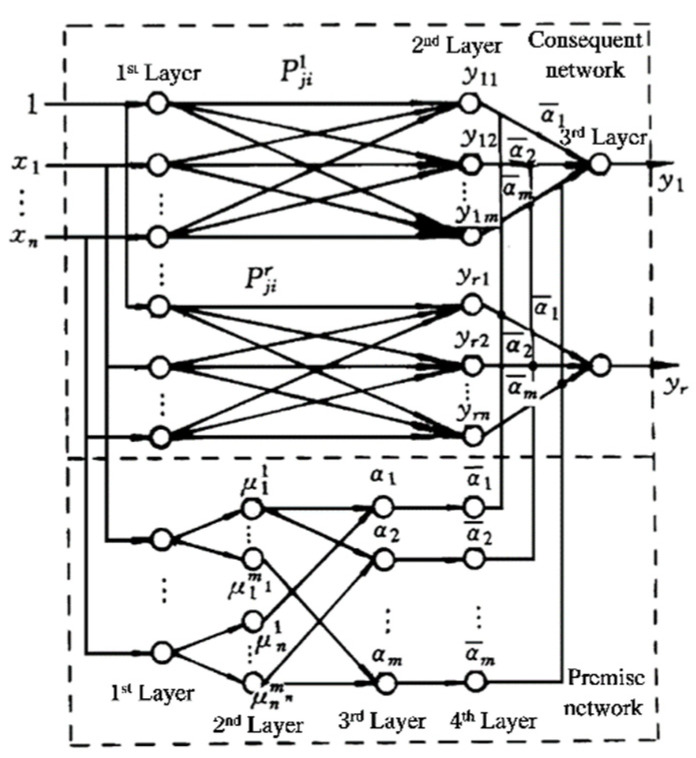
Fuzzy neural network structure diagram of T-S model.

Due to the complexity, nonlinearity and strong coupling of the interaction between wind, waves and ship, it is impossible to establish a clear mathematical model to reflect the real-time movement state changes of the ship [27]. In order to obtain more accurate speed and attitude changes, this paper designs an ANFIS based on the double-input single-output T-S model [28,29]. The structure of the fuzzy neural network of the T-S model is shown in Figure 8. The network consists of a premise network and consequent network. The premise network is used to match the premise of fuzzy rules, and the consequent network is used to generate the consequents of the fuzzy rules. The learning algorithm of ANFIS will not be detailed here.

In this paper, the first layer of the premise network comprises two input variables, which are the accelerations in Equation (17) and Equation (19), respectively. The membership function type of the input variables is Gauss, the number of membership functions is 5 and 7, respectively, and the output variable type is linear. The second layer is used to calculate the membership function of the input variables. Each node in the third layer represents a fuzzy rule, which is used to match the premise of the fuzzy rule and calculate the fitness of each rule. The fourth layer is used to achieve normalization. In the consequent network, the first layer is the same as the antecedent network. The second layer is used to calculate the consequent of each rule. The third layer is used to calculate the output of the ANFIS system, and the output is the speed change of the ship.

**Figure 9:**
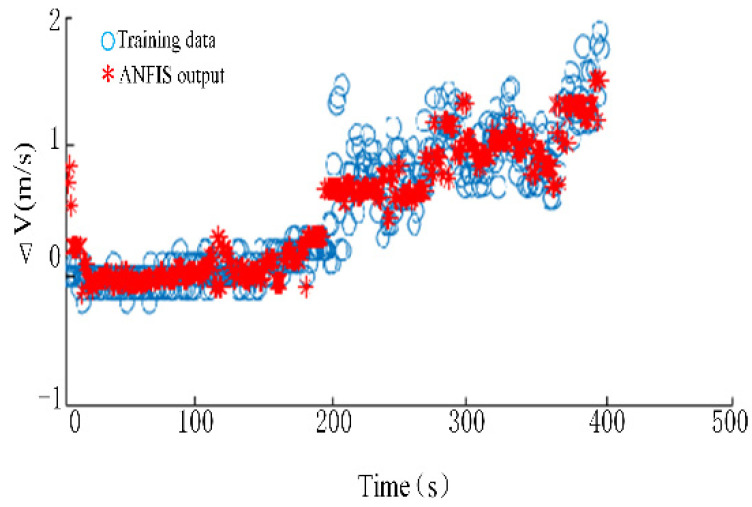
Comparison chart of training data and ANFIS output.

Taking the measured data of the ship as the training set, the input of the training set is the measured wind speed, wind direction, wave height and wave direction. The output is the speed change, which is the change in current speed subtracted from the ship’s speed change. In order to reduce the influence of propeller and rudder forces, the measured data of the ship when sailing straight at a constant speed are selected as the training data. Because the ship is in dynamic equilibrium under this motion state, the changes in its speed and attitude can be seen to be caused by time-varying wind, current and waves. The training result of ANFIS is shown in Figure 9. After training, the average error between the system output and the training data is 0.2005, meeting the error tolerance.

## 4. Simulation Analysis and Comparative Verification

Due to the time-varying nature and randomness of wind, current and waves, the time-varying non-uniform wind, current and wave field constructed in this paper cannot be consistent with the wind, current and waves encountered by the ship in actual navigation, and its impact on the ship’s motion state cannot be consistent with the measured data. In order to verify the rationality and superiority of this method, the following simulation strategies are formulated:

(1) Based on the ship trajectory generator designed in this paper, the ship motion parameters without wind, current and wave interference are generated, and then the wind, current and wave interferences are superimposed to compare and analyze whether the change in motion parameters is reasonable;

(2) We select a period with a relatively large disturbance of wind and waves from the measured data; take the actual measured wind speed, wind direction, wave height and wave direction as the input of ANFIS; generate the corresponding speed change; and then add it to the ship trajectory generator in this paper to observe whether the accuracy of the main motion parameters has been improved.

### 4.1. Simulation Analysis

The trajectory generator described in this paper generates ship motion parameters, sets the level of wind, current and waves and generates corresponding interferences in combination with the wind, current and wave fields. The initial information, maneuvering state and detailed information of wind, current and waves are shown in Table 4, Table 5 and Table 6.

**Figure 10:**
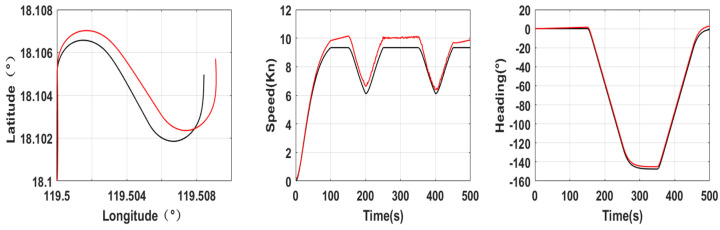
Comparison of simulation data without and with interference.

The simulation results are shown in Figure 10. The black lines and red lines in the figure represent the simulation results without interference and with interference, respectively.

It can be seen from Figure 10 that after the interference is added, the movement of the ship changes as follows:

(1) The track obviously deviates to the east and north;

(2) The speed generally increases, slightly increases before steering, and then increases as a whole;

(3) The overall trend of the heading angle changes little—it decreases slightly after the first steering and increases slightly after the second steering.

According to the information in Table 4, Table 5 and Table 6, the above changes are analyzed as follows:

(1) The wind direction is 45° north by east, and the flow direction is 90° north by east. Therefore, under the combined action of wind and current, the ship’s track will naturally drift to the east and north.

(2) In the wind, current and wave interference considered in this paper, the current has the greatest impact on the ship’s speed. Table 6 shows that when the ship does not start to steer, the current direction is almost perpendicular to the ship’s moving direction, so it has little impact on the speed. However, the speed caused by wind has an influence on the ship’s speed, so the ship’s speed rises slightly before steering. After the first steering, the current begins to exert an influence on the direction of the ship’s motion, which causes the ship’s speed to rise.

(3) As the wave level is small, it has little influence on the heading angle. It can be seen from the wind direction that the wind will cause the ship’s heading angle to change to the north by east direction. The ship will move southward after the first steering, so the wind will reduce the ship’s heading angle. After the second steering, the ship will move northward, so its heading angle will increase.

Therefore, the above changes are reasonable after the interference, which shows the rationality and feasibility of the wind, current and wave interference model proposed in this paper.

### 4.2. Simulation Comparison Verification

In order to verify the accuracy of ANFIS, based on the ship trajectory generator presented in this paper, the ship motion parameters are generated using the same initial state and maneuvering state. We compare the accuracy of the ship motion parameters with and without wind, current and wave interference. The initial information and the maneuvering status of the ship are shown in Table 7 and Table 8.

**Figure 11:**
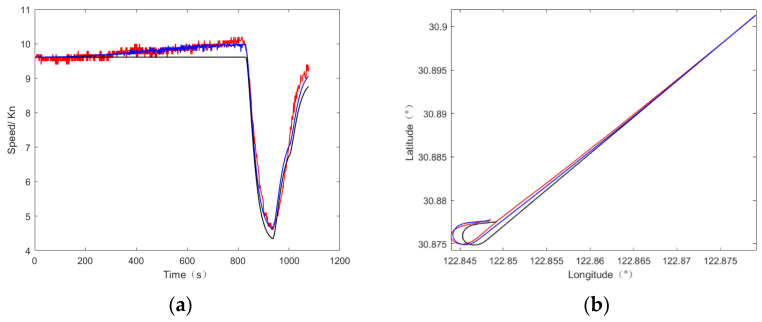
Comparison chart with and without interference and measured data. (**a**) Speed comparison chart (**b**) Track comparison chart.

The comparison results of the simulated speed and track are shown in Figure 11. The red line in the figure is the measured data; the blue line is the data generated by the ship trajectory generator with wind, current and wave interference; and the black line is the data generated by the ship trajectory generator without wind, current and wave interference.

It can be seen from the above figures that, compared with the ship trajectory generator without wind, current and wave interference, the accuracy of the speed and track after adding ANFIS is significantly improved, especially in the constant speed stage, where the change in speed is more consistent with the measured data; the specific accuracy and RMSE values are shown in Table 9. Since the accuracy of the position is obviously improved, if the same threshold value as in Table 3 is adopted, the position accuracy will be 100%, so the threshold value of the position listed in Table 9 is adjusted to 35 m.

The above simulation results show that the method proposed in this paper can better simulate the impact of the interference of wind, current and waves on ship motion, and they also verify the advantages of ANFIS.

## 5. Discussion

The wind, current and wave field models constructed in this paper can reflect the characteristics of wind, current and waves. However, due to the randomness and time variability of wind, current and waves, there is a lack of effective evaluation and verification methods, and it is difficult to compare and verify the model with the measured data. Therefore, it is necessary to obtain appropriate evaluation methods for wind, current and wave models.

The force of ship motion at sea is complex and the coupling degree of wind, current and wave interference is high. The ANFIS designed in this paper takes the data under the condition of straight sailing at a constant speed as the training data, and the interference of wind, current and waves under the condition of variable speed or steering needs further research.

## 6. Conclusions

In this paper, a new mathematical model of a ship trajectory generator is established; the interference of wind, current and waves with ship motion is analyzed, and an adaptive neuro fuzzy inference system for wind, current and wave interference is designed. After simulation analysis and verification, the following conclusions are obtained:

(1) The motion parameters generated by the ship trajectory generator proposed in this paper are more accurate than those generated by the typical trajectory generator. After the wind, current and wave disturbances are superimposed, the accuracy is further improved. The ship trajectory generator has certain significance for the simulation testing and algorithm research of a ship’s strapdown inertial navigation system.
(2) The wind, current and wave fields constructed in this paper can reflect the randomness and time variability of wind, current and waves on the sea. Combined with the designed ANFIS, we can simulate the corresponding interference, which has certain significance for the research of ship motion under wind, current and wave conditions.
(3) Compared with the traditional mathematical model of ship motion, the trajectory generator described in this paper does not require complicated hydrodynamic parameters, has low modeling requirements and has the advantage of high simulation accuracy, and it can be used in the field of navigation simulators.

## Figures and Tables

**Table 1:** Initial information table.

Lng (°)	Lat (°)	*v* (kn)	Heading (°)
123.05516	31.06247	8.7	239.5

**Table 2:** Maneuver state table.

Time (s)	500	120	325	35	50	120
State	Linear	Linear	Linear	Steering	Steering	Steering
Δ*V* (kn)	0	8.2	0	−0.8	0.8	2
*δ* (°)	0	0	0	20	15	−20

**Table 3:** Accuracy and RMSE comparison results.

Parameter		Pos (m)	*v* (m/s)	Heading (°)
Threshold		70	0.1	1
Accuracy	Typical	52.09%	64.96%	94.00%
	New	85.48%	68.70%	94.96%
RMSE	Typical	72.4141	0.2743	2.6821
	New	50.8821	0.2441	2.1743

**Table 4:** Ship’s initial condition.

Lng (°)	Lat (°)	*v* (kn)	Heading (°)
18.10	119.50	0	0

**Table 5:** Maneuver state.

Time (s)	100	50	50	50	100	50	50	50
State	Linear	Linear	Steering	Steering	Linear	Steering	Steering	Linear
Δ*V* (kn)	10	0	−5	5	0	−5	5	0

**Table 6:** Wind, current and wave conditions.

Wind		Current		Wave	
Level	Dir	Speed	Dir	Level	Dir
4	45°	1 m/s	90°	2	45°

**Table 7:** Ship’s initial condition.

Lng	Lat	Speed	Heading
122°52′45″	30°54′05″	9.6 kn	225.9°

**Table 8:** Maneuver state.

Time (s)	830	106	64	77
State	Linear	Steering	Steering	Linear
Δ*V* (kn)	0	−5.4	2.8	1.8

**Table 9:** Accuracy and RMSE comparison results.

Parameter		Pos (m)	*v* (m/s)
Threshold		35	0.1
Accuracy	Without ANFIS	42.99%	55.62%
	ANFIS	88.12%	87.00%
RMSE	Without ANFIS	68.9173	0.1641
	ANFIS	23.4941	0.0948
